# Patient co‐design of digital health storytelling tools for multimorbidity: A phenomenological study

**DOI:** 10.1111/hex.13614

**Published:** 2022-09-27

**Authors:** Marissa Cummings, Jay Bradley, Gemma Teal

**Affiliations:** ^1^ The Innovation School The Glasgow School of Art Forres UK; ^2^ The Innovation School The Glasgow School of Art Glasgow UK

**Keywords:** co‐design, digital, multimorbidity, narrative medicine, participatory visual methods, reflexive interviewing

## Abstract

**Background:**

Interest in both narrative medicine and electronic health records has increased over the past 30 years. However, electronic health records are mainly written by and for clinicians, and the patient narrative and voice are not incorporated. Recent studies within the United Kingdom have indicated that there is a need to incorporate patient stories into health records, to improve quality and continuity of care. This is particularly important when treating people with multiple long‐term health conditions (multimorbidity), whose health stories can be particularly complex.

**Objective:**

To understand the goals and requirements of people with multimorbidity for digital health storytelling tools.

**Methods:**

The methodology uses narrative within a phenomenological approach to inform a process of co‐design.

**Results:**

The findings indicate that people living with multimorbidity would use health storytelling tools to understand and reflect on their journeys, convey their experiences to others and advocate for themselves against scepticism.

**Conclusion:**

Outputs from the project give insight into the lived experience of multimorbidity, as well as understanding the goals of people living with multimorbidity for using health storytelling tools as part of treatment and self‐management. Future research could explore other areas such as collaborative health storytelling or the technical implementation of tools.

**Patient or Public Contribution:**

Five adults with multiple long‐term conditions participated in the project, and research was carried out in three stages. First, semistructured interviews were used to understand each participant's health story. Second, each participant worked with the researcher to co‐design a visual representation of their story. Finally, digital prototypes based on their health story were reviewed with each of the participants.

## 1 INTRODUCTION

Over the past 30 years, there has been a growing interest in the incorporation of patients' health stories into treatment as part of person‐centred care.^1^ This is part of an overall trend which recognizes health and well‐being as part of the wider context of an individual's life.^2^ The field of narrative medicine, for example, includes such activities as storytelling by healthcare providers and patients, journaling and active listening and reflection on the stories of others.^3^ Research has shown that the use of narrative in care has tremendous benefits for people with health conditions: improving their mental health and well‐being,^4^, ^5^ improving the quality of their care^3^ and also promoting cooperation between patients and healthcare professionals.^6^

At the same time, our world has become increasingly digital, and healthcare has been no exception to this trend. In Scotland, there is an acknowledgement that people now expect health services to be digitally enabled.^7^ Despite this, efforts have largely focused on allowing patients to view their health information rather than allowing them to include their own voices. While this may be suitable for occasional service users, people with long‐term conditions may prefer to adopt a more empowered approach to their care.^8^

People living with multiple long‐term conditions (multimorbidity) may particularly struggle in a healthcare system which is primarily designed for the treatment of single, acute conditions.^9^ The numbers of people with multimorbidity are also steadily rising, making their treatment a pressing concern in healthcare.^10^, ^11^ The interactions between different conditions mean that the health stories of people with multimorbidity are necessarily complex, encompassing a wide spectrum of contexts beyond the clinical setting.^12^ Understanding these stories is therefore a crucial part of treatment.^13^

Within the past 5 years, several projects within the United Kingdom have identified a need for health stories to be included alongside patients' electronic health records, as well as exploring in a limited way how such tools might look and function.^14^, ^15^, ^16^ This project builds on this previous work, but also looks at how health stories can be used more broadly: as a means of creative expression and personal empowerment, and as a tool for people to shape and reflect on their care. Adopting a phenomenological approach of dialogic, relational research,^17^, ^18^ participants worked closely with the researcher in cycles of co‐creation, narrative analysis and reflexive digital design practice. The work was carried out as a year‐long Masters of Research project, funded by Scotland's Digital Health & Care Innovation Centre. The aim of the project was to understand the attitudes of people with multimorbidity towards health stories, and their goals for using health storytelling tools.

### 1.1 Reflexivity statement

The researcher on this project has lived experience with long‐term health conditions, and professional background as a digital interface designer and developer.

## 2 MATERIALS AND METHODS

**Figure 1:**
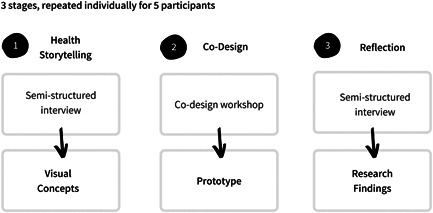
Methodology comprised of three cycles of fieldwork and analysis

The fieldwork used reflexive, semistructured interviews to drive an overall process of co‐creation with the research participants (Figure 1). Each participant worked with the researcher individually, meaning that no participant ever met another. Fieldwork was organized into three cycles of activity. Each cycle commenced with a participant interview. Narrative analysis^19^ was used to extract the overarching themes in dialogue with participants, which then informed a reflexive practice of digital interaction design by the researcher. The output from the prototyping was then carried forward to form the foundation of the next cycle. The final outputs of the work were individual digital prototypes which reflected the health stories of each participant, alongside overall findings on their perspectives on the uses of a health storytelling tool.

### 2.1 Participant recruitment

Participants in the project were adults with multimorbidity residing in Scotland. Following the phenomenological approach, the project recruited five participants, with multiple interactions with each participant. Participants were recruited via patient support groups for conditions with a high incidence of multimorbidity. Interested participants were then directed to a webpage where they could review the project information (Supporting Information: Appendix A). One participant (Hazel) found out about the project through word of mouth. All participants are identified by assigned pseudonyms throughout.

The final cohort was comprised of one man and four women between the ages of 31 and 53 and included the following conditions: borderline personality disorder, chronic migraines, depression and anxiety, early menopause, fibromyalgia, irritable bowel syndrome, myalgic encephalomyelitis (ME), polycystic ovary syndrome, posttraumatic stress disorder, postural tachycardia syndrome, psoriatic arthritis, Raynaud's disease and relapsing‐remitting multiple sclerosis.

### 2.2 Co‐creation

The entire dialogic research process within this project can be viewed as a form of co‐creation, defined as ‘any act of collective creativity, i.e., creativity that is shared by two or more people’.^20^ The co‐creation took two forms. First, the research approach allowed ‘dialogic co‐creation’^18^ of the research findings, which emerged organically from the relationship which developed between the researcher and the participant. Second, the project also explicitly used the method of co‐design,^20^ a process in which the researcher acts as a facilitator, creating toolkits that the participants can use to design their own preferred outcomes. The focus in co‐design is therefore not on what participants *say* but on what they *make*, making co‐design a useful way of gaining insight into designing for the future.^21^ These activities also incorporated elements of elicitation, in that the co‐designed artefact also served as a focus for discussion (described further in the following section).

### 2.3 Reflexive interviewing and participatory visual methods (PVMs)

Reflexive interviews are semistructured, conversational and episodic, with periods of reflection by both parties in between.^22^ This approach allows the participant to verify the researcher's analysis, creating a ‘shared intelligibility’^23^ which adds further rigour to the research. Interviews for this study were carried out remotely via Zoom during the COVID‐19 lockdown and were recorded with the participants' consent. Each participant was interviewed three times over a 2‐month period (Supporting Information: Appendix B).

PVMs^24^ have been praised as giving participants an empowering voice within the research process. In this project, PVMs were used alongside the interviews to provide a focus for the discussion. The first interview used an adaptation of Marini's health storytelling prompts,^25^ which have been developed to elicit a comprehensive health story from participants using only basic English. This was combined with a form of mixed object elicitation and photovoice,^26^ in which participants were asked to select and photograph an object which represented their health to discuss with the researcher. In the second interview, participants were shown a variety of visuals, some of which had been created by the researcher through reflexive analysis. This was then used as graphic elicitation^27^ during the interview. In the third interview, participants were shown an interactive digital prototype of a health storytelling tool based on their story. Again, this was used as graphic elicitation.

### 2.4 Analysis

The analysis took place following each stage of the fieldwork (Supporting Information: Appendix C).

Narrative analysis^19^ examining the holistic meaning and form was first used to develop an understanding of each participant's story. Following this, further analysis was done to pull out themes from the findings, first within each individual story, and then across all the participants. At each stage, a written summary of the findings was taken back to the participants following a dialogical approach.^17^ This allowed participants to review and dynamically consent^28^ to their inclusion in the final output, as well as validate the researcher's analysis.

Using the findings from the narrative analysis, the researcher designed digital images and interactive online prototypes representing a health story tool for each participant. These were used during the second and third interviews as another form of graphic elicitation. Although the design of the prototypes was a creative process, within this study it was also used as a form of analytical practice. This follows the argument of Creative Analytical Practices^29^ whereby analytical output may take creative forms such as artwork or poetry.

## 3 RESULTS

The outputs of the project were individual prototypes for health storytelling, co‐designed with each of the research participants, as well as findings on patient requirements for a health storytelling tool. The following sections summarize the findings for each participant along with a description of their individual prototype. These were developed through the analysis done in dialogue with the participants. The final section summarizes the overall findings across all participants, which was done separately by the researcher after the conclusion of the fieldwork.

### 3.1 Hazel

**Figure 2:**
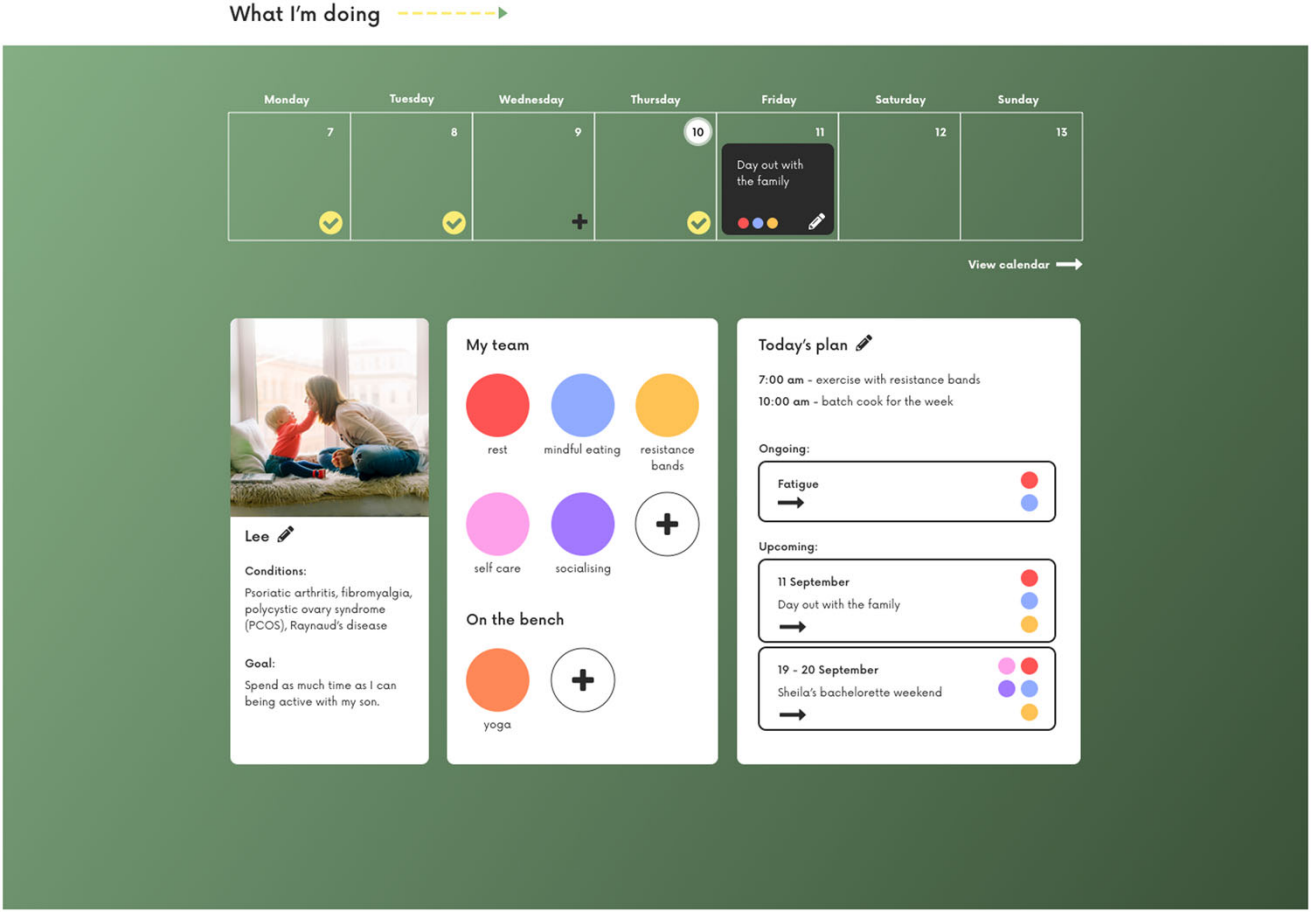
Hazel's prototype—health storytelling based on self‐management, using a sport analogy

Hazel's goal was to use self‐management to control her condition so that she could live her life as normally as possible. For her prototype, we used a sport metaphor to demonstrate a health story which is told *through* self‐management activities (Figure 2). Individual self‐management activities take on the role of *players* who can be selected by Hazel to form a *strategy* which will help her overcome particular obstacles. Strategies can be ongoing (e.g., dealing with daily fatigue) or short‐term (e.g., planning for a social event).

Hazel can also record *player profiles* in the prototype which record detailed information about each activity and the costs and benefits of employing a particular activity. This reflects themes from Hazel's interviews around developing a base of evidence on what works for her to share with her healthcare team. Because Hazel's first diagnosis came at an early age, she felt that this created a power imbalance in her care relationships which evidence helped her address.

### 3.2 June

June often feels the need to spend time alone, a tendency that she attributed to her mental health conditions. However, emotional connections with others were also important to her, and she viewed her support network as critical for self‐managing her conditions. She wanted to be able to share parts of the story with others, but only in a controlled way.

**Figure 3:**
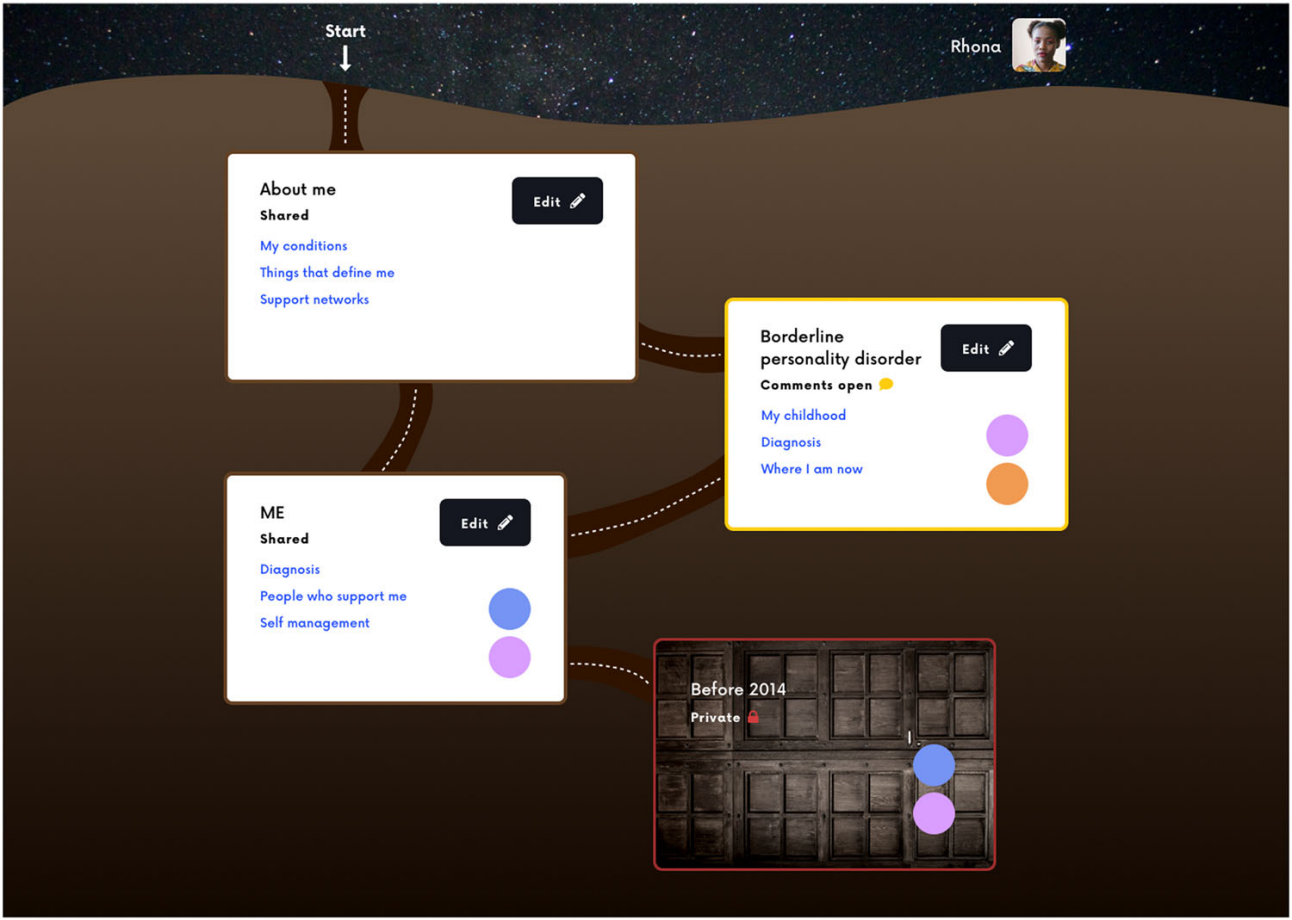
June's prototype—a burrow of stories, with an emphasis on privacy

June's love of enclosed spaces, combined with her desire to control and separate the information she presented to others, resulted in the idea of a prototype based on a burrow with individual story *caves* (Figure 3). Each story cave could be composed of separate episodes grouped together under a single heading. Caves could contain different groups of collaborators, playing on ideas of togetherness and also moving through a physical space.

Privacy was very important to June, so we included multiple ways for her to control content sharing. At the default level, content is simply visible to others. June could also have *private stories* which are only visible to herself or shared with trusted friends and family members. On the other side, June may have parts of her story where she is actively seeking input or advice from others. The prototype also included a way for June to *seek guidance* by sending out a call for help from collaborators—an idea inspired by her interest in spiritual guidance.

### 3.3 Alison

The primary theme of Alison's story is *memory*. It is important to Alison that she remembers everything that has happened to her, and that she accumulates as many good memories as she can during her lifetime. She feels that she has a ‘finite amount of time’, as her primary condition, multiple sclerosis, could progress to the point where she is no longer able to do things.

**Figure 4:**
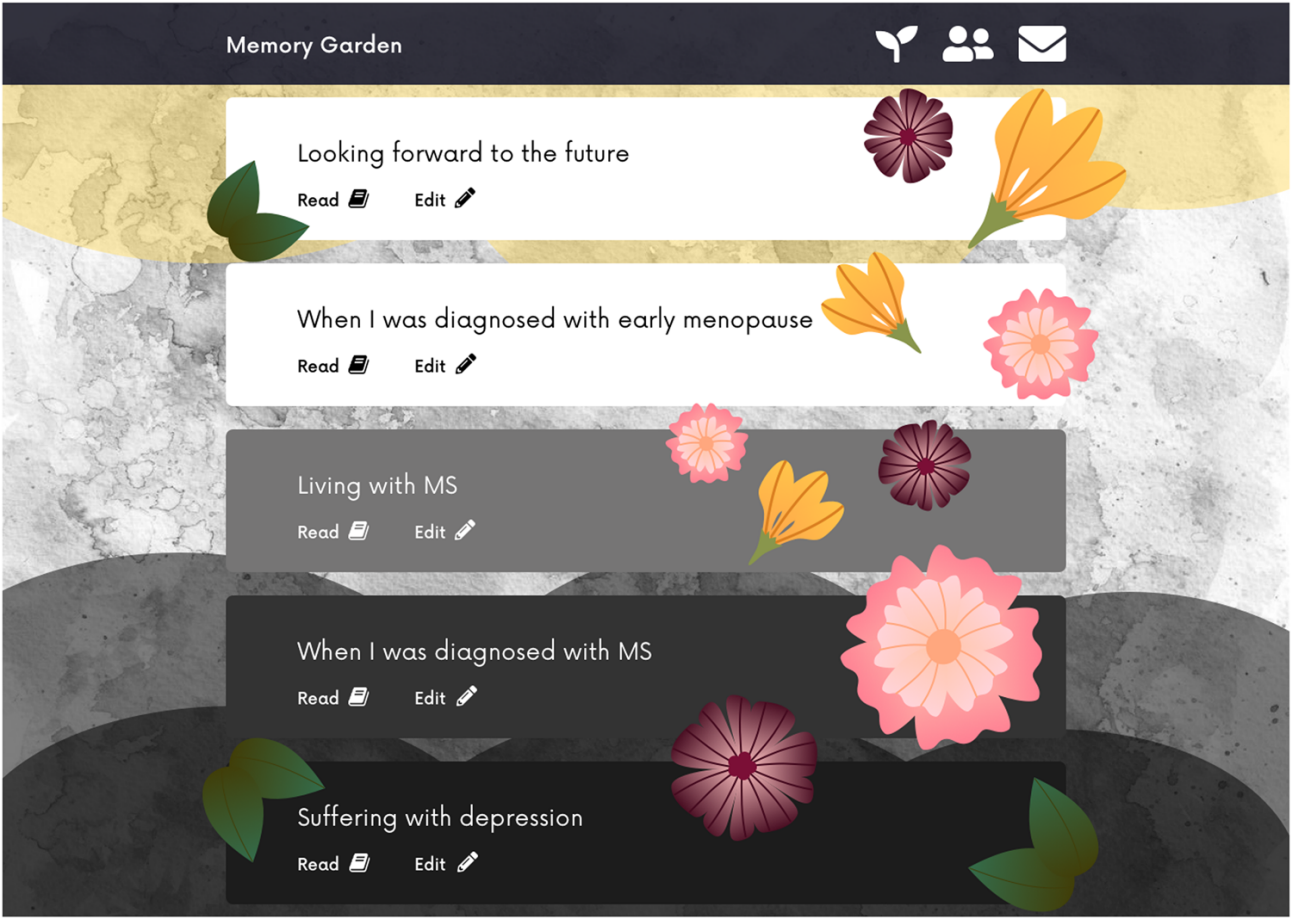
Alison's prototype—a memory garden of light and shadow

Alison's story contained elements of deep sadness, but also humour and joy. These came together in the prototype in a concept of highlights and shadows, providing a visual representation of Alison's emotional state over the course of the story (Figure 4). Alison could set a *mood* for each story which would generate the light/dark mode on the overall view.

Because Alison generally told her story in vignettes, her prototype broke her story down into individual events mapped out chronologically. In the final design, we also incorporated the idea of a *memory garden*. Memories were represented as flowers which can be linked to stories in Alison's life.

Alison wanted friends to be able to communicate with her about her story, but only in a general way. We represented this by allowing friends to post emoji reactions on her story (e.g., a heart), and also message her directly using a contact form.

### 3.4 Gillian

Gillian discussed how difficult she found it to ‘stay on course’ with her treatments for secondary breast cancer. This included understanding her feelings towards her health and that of those around her, as well as keeping track of treatment options.

**Figure 5:**
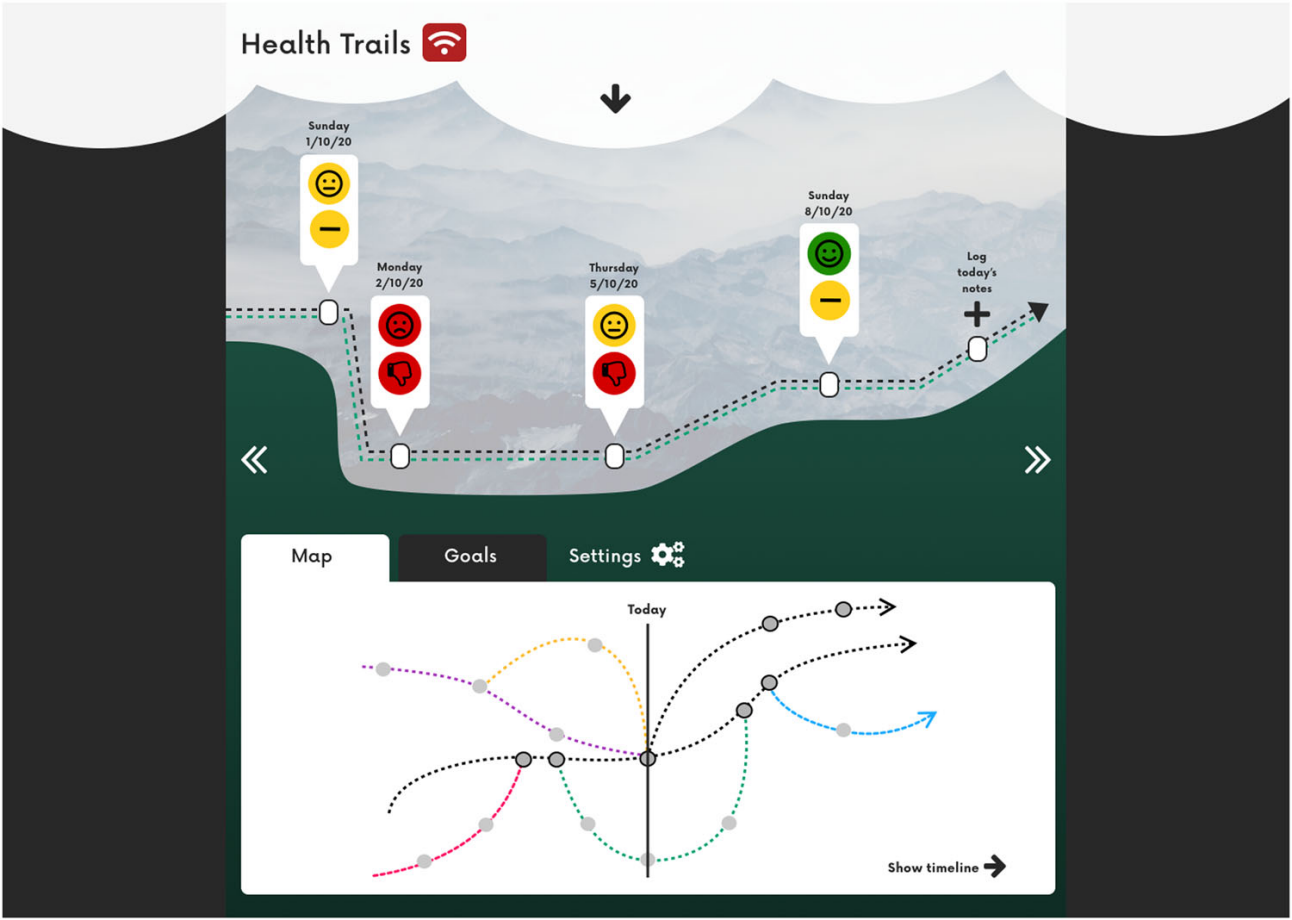
Gillian's prototype—a treatment trail map

Using Gillian's interest in outdoor activities, we came up with the idea of a prototype based on a *trail map* which mapped out different treatment routes (Figure 5). The map would clearly state Gillian's overall goals, as well as the potential outcomes of each treatment, helping Gillian to determine whether a particular treatment route met her goals. The concept of peaks and valleys illustrates how one's condition progresses over time. Gillian could also record *trail notes* to document the daily status of her physical and emotional states as well as recent activities. During acute periods of care, Gillian could enable a Follow Me feature which would allow interested friends and family to get updated on her treatment schedule so that they can easily check in with her.

As a person with a life‐threatening condition, Gillian found it too overwhelming at times to know the details of what was happening. We represented this using the concept of *cloud cover*, a screen which can be pulled up or down on the main page. Using this would let Gillian control how much detail she sees, and how far into the future her map will extend. When cloud cover is enabled, it could also flag to friends and healthcare professionals that Gillian does not want to have detailed discussions about her health.

### 3.5 John

The genre of John's story is probably closest to Arthur Frank's idea of the *quest*,^30^ in that he has a specific goal: that one day, there will be a treatment or cure for his primary condition, ME. A reoccurring theme in our discussions was *hope*. John felt that it was easy to ‘burn up’ one's hope in the search for potential treatments, and that this must be carefully cultivated and conserved for the future.

**Figure 6:**
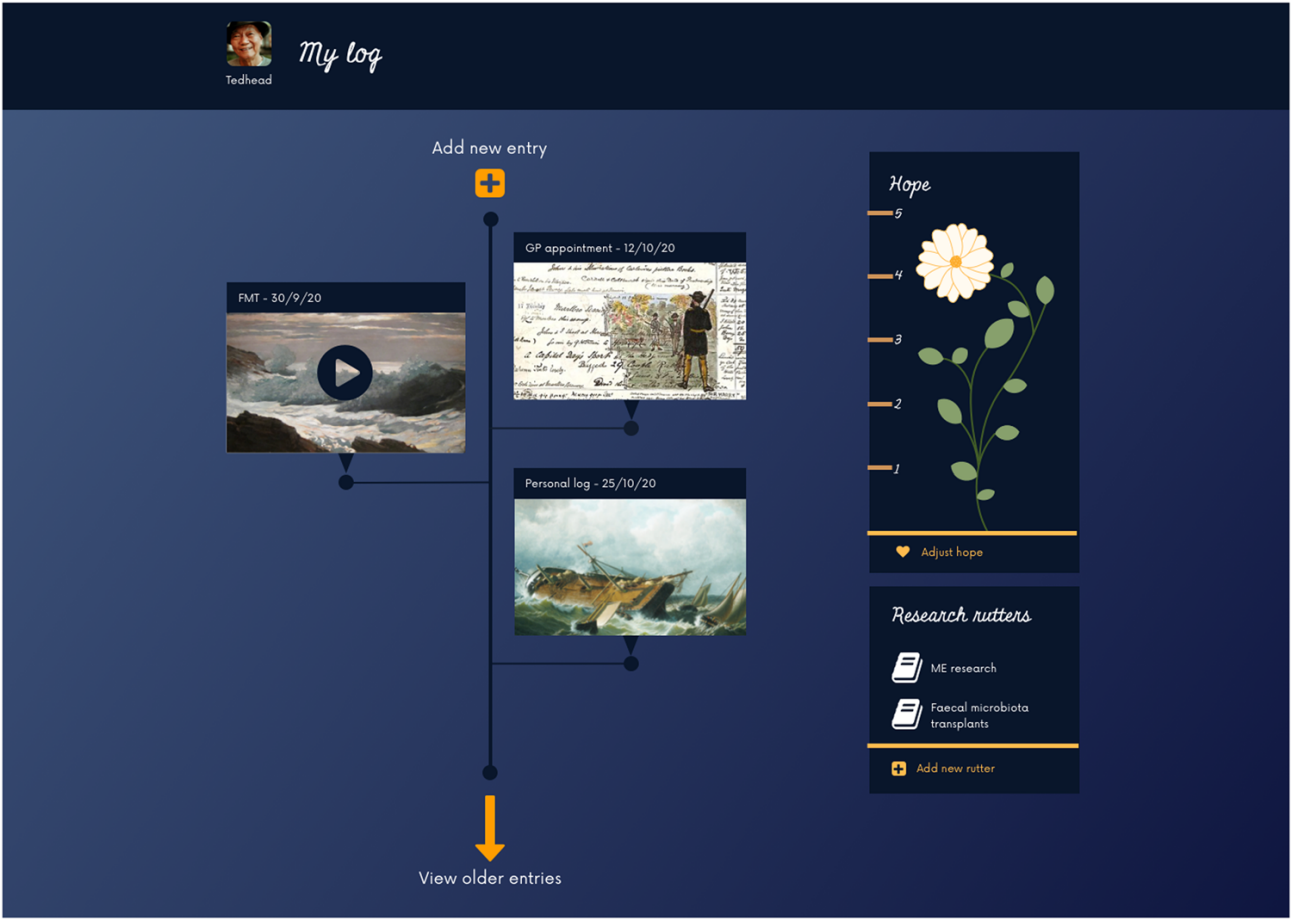
John's prototype—a ship's log of a health voyage, with a nautical theme

Because of John's interest in sailing, we came up with the idea of portraying his health story as a *ship's log* (Figure 6). As John sometimes struggles with fatigue, entries could be video recorded if he is feeling too tired to type. Treatment events, such as appointments, could also be appended with a log entry to describe his thoughts on what happened.

John's ideas about hope were represented within the prototype as a *hope flower*. The flower has several increments which can be adjusted or up or down to give a simple visual representation of John's current hope level. This could then be used for personal reflection or shared with others.

Because John does considerable research on his conditions, the concept also includes the idea of *research rutters*, based on the idea of a ship's rutter. These are areas where John could record notes and resources, such as treatment options. The rutters can also be linked to log entries as a loose form of citation. A healthcare professional could use these to learn about something John is interested in and to understand John's approach towards his illness (as in Kleinman's idea of explanatory models^31^). He is very involved with patient advocacy, so we also considered how the rutter might operate as a joint work with several contributors.

### 3.6 Overall findings

Participants identified three primary audiences for a health storytelling tool: family and friends, healthcare professionals and the participants themselves (Table 1). They also identified a number of goals/uses for the tool (Table 2). These can be distilled into three overarching goal categories:

(1) Goal 1 (G1): Seeing the big picture;
(2) Goal 2 (G2): Conveying the illness experience;
(3) Goal 3 (G3): Challenging scepticism.

**Table 1:** Audiences and collaborators identified by participants

	Hazel	June	Alison	Gillian	John
Family/friends	X	X	X	X	X
Healthcare professionals	X	X	X	X	X
Self	X	X	X	X	X
Disease‐mates	?	?	X		X
Alternative healthcare professionals	X			X	
Co‐workers	X	X			
Official bodies (e.g., government)		X			X
Advocacy groups					X

*Note*: Blank, not important; ?, sometimes important; X, important.

**Table 2:** Goals of a storytelling tool identified by participants, organized by overall category

	Hazel		June		Alison			Gillian	John	
**G1: Seeing the big picture**										
G1.1 Remembering	?		X		X			?	?	
G1.2 Self‐reflection	X		?		X			X	X	
G1.3 Daily self‐management	X							X	?	
G1.4 Understanding one's conditions and treatments								X	X	
**G2: Conveying the illness experience**										
G2.1 Supporting communication			X		?			X	X	
G2.2 Getting support	X		X		X			X		
G2.3 Inviting alternate perspectives/sense‐making			X					X		
**G3: Challenging scepticism**										
G3.1 Evidencing what has happened	X				X				X	
G3.2 Sharing knowledge			?		X				X	
G3.3 Education and advocacy								?	X	

*Note*: Blank, not important; ?, sometimes important; X, important.

The prototypes also show what types of functionality a health storytelling tool might require (Table 3). These can be roughly divided into two categories: the ability to record information about oneself, and the ability to share information with others.

**Table 3:** Features/functionality of a storytelling tool identified by participants, organized by category

	Hazel	June	Alison	Gillian	John	
F1: Information about oneself						
F1.1 Mental health tracking	X	X	X	X	X	
F1.2 Written accounts (journaling)		X	X	X	X	
F1.3 Visual representations and media		X	X		?	
F1.4 Record of self‐management activities	X			X	?	
F1.5 Goals				X	X	
F1.6 Physical health tracking	X			X	
F1.7 Treatments and outcomes				X	X	
F2: Sharing with others						
F2.1 Privacy and access controls		X	X	X	X	
F2.2 Discussion/messaging features		X	X		?	
F2.3 Story export feature	X	X		X		
F2.4 Knowledge/research recording		?	?		X	
F2.5 Features supporting collaborative advocacy work					X	

*Note*: Blank, not important; ?, sometimes important; X, important.

#### 3.6.1 Goal 1: Seeing the big picture

Participants wanted the ability to look back and remember what had happened to them (G1.1), either for self‐reflection (G1.2) or to celebrate ‘small wins’ (Hazel). This included both positive and negative events, as embodied in Alison's *light and dark memory garden*, and was viewed as important for mental health.

It's easy to forget things … [T]he memory wall … is a fantastic idea. Because it's not all bad … there is some good bits in there as well. It's not all rubbish. (Alison)

Recording details of daily self‐management (G1.3) was important to many of the participants, and they wanted to be able to build an understanding of trends and patterns. Being able to see how activities affected one's health, and plan positive activities in, was considered useful.

I might be doing the same things consistently, like working out, mindful eating … But then, I might still need those same … players when I'm going to have that day out, as well, but just factoring in more rest. … Seeing that planned in definitely does help. (Hazel)

Appointments with healthcare professionals tended to be infrequent, so remembering details of daily activities was also practically useful. Participants related this to evidencing (G3), saying that they felt they sometimes needed to ‘prove’ what they had been doing to professionals.

Recording information was also important for participants to understand their conditions and ongoing treatments (G1.4). This was particularly noted by Gillian (who was undergoing numerous treatments for cancer) and John (who felt dissatisfied with the treatment options suggested by professionals).

#### 3.6.2 Goal 2: Conveying the illness experience

Participants identified many situations in which a health storytelling tool could act as a useful aid to the conversation (G2.1). First, participants found it hard to initiate deeper conversations or express concerns even when they were being asked about these directly. A tool could facilitate conversations by providing details about their health, while still giving them control over how much to show collaborators. Second, participants spoke about the difficulty of articulating events which affected their mental health. A visual tool could support this by giving them more abstract ways to convey emotions. For example, June spoke about wanting to include images and animations in her story.

There were also scenarios in which participants might want to give information about their health without speaking to someone directly. This could be because of storytelling fatigue,^14^, ^30^ because of an uncomfortable topic, or simply feeling too unwell.

I like that I can message people as well, I really like that aspect of it … I think, for me … everything's got to seem like I'm ok. I find it really hard to ask for help … I quite like that idea of just sending a message and people can access it … For me that would be extremely beneficial. (June)

On a practical level, participants wanted to be able to coordinate communication among healthcare professionals. This included being able to promote continuity of care by quickly giving new professionals an idea of their history, personality and concerns.

Conveying the nature of the illness experience was also important for participants in gaining support from others (G2.2). Whether someone ‘got it’ made a big difference to the level of support offered.

[Secondary breast cancer is] … such a devastating diagnosis on the one hand, on the other hand, if you're going to have any life‐threatening condition it's a good one to have because people get it. Or think they get it. (Gillian)

Finally, the act of discussing one's health with others could also act as a form of reflection. Participants discussed wanting to make sense of what was happening by gaining outside perspectives from friends, family and disease‐mates (G2.3).

#### 3.6.3 Goal 3: Challenging scepticism

Participants said they often had to challenge scepticism about the nature of their conditions, even when working with healthcare professionals. A storytelling tool was seen as being a good way of evidencing one's lived experiences (G3.1), related to the goals of *remembering what has happened* (G1.1) and *getting support* (G2.2).

There was also an aspect of education, where participants wanted to share an understanding of their conditions with others (G3.2). Sharing with professionals was viewed as being particularly important when their opinions diverged from the patient.

Communicating with medical professionals is by far the hardest nut to crack … because the entire ‘social contract’ with your doctor is predicated on them having knowledge and power and the patient having an unmet need. When challenging their misconceptions about illness the patient challenges this whole model: the patient has knowledge … but not power; the doctor may or may not have an unmet need—the need to be better informed and transform their practice. (John)

Participants also related knowledge sharing to patient advocacy (G3.3). Stories were seen as something which could be used to advocate for change—both within their immediate support circles and on a societal level.

### 3.7 Desired functionality

The functionality of a health storytelling tool identified by participants can be divided into two categories:

F1. Tracking information about oneself;

F2. Sharing with others.

Within the first category (F1), the most important feature was tools for tracking mental health (F1.1), which was mentioned by every participant. In contrast, only two participants mentioned tracking physical health (F1.6), which may be due to the prevalence of existing tools for this purpose.

Privacy (F2.1) was the most important feature in the second category (F2). In some cases, participants wanted information to be visible only to themselves. While all the participants wanted to share their stories with others, they also wanted to limit how collaborators could interact with them (F2.2 and F2.3)

## 4 DISCUSSION

The findings from the project show that the health stories of multimorbidity are complex, with implications far beyond a clinical frame.^32^ Although ostensibly stories about health, the stories that participants told were really about their lives. Much of the impact that participants described sat outside of the normal scope of clinical treatment—for example, John mourning the loss of his boat and his ability to sail. Every participant had experienced difficulties with mental health, not uncommon among people with multimorbidity,^33^ and there was a desire to have this emotional dimension reflected in their health records. Participants also rarely spoke of their conditions separately.^13^ Even when conditions were apparently unrelated, participants perceived them as part of a continuous experience. This suggests that tools for health stories need to be open‐ended, modular and easily extendable to accommodate many different modes of storytelling.

The prototypes which were developed with participants also demonstrated a desire for nuance in recording their experiences. For example, Hazel's prototype with its ideas of *self‐management players* with strengths and weaknesses goes beyond simpler, commonly used frameworks for describing the illness experience such as Spoon Theory,^34^ which uses spoons as a finite unit for measuring energy levels.

### 4.1 Health and care relationships

Participants wanted to use their health stories to build better relationships with people in their support networks by promoting, as one participant expressed it, ‘mutual understanding’. A health storytelling tool could promote empathy and understanding amongst a person's support networks, simply by giving others insight into the illness experience—particularly important to stigmatized conditions.^35^

The participants' stories showed that collaborators (e.g., friends, family or healthcare professionals) within a health storytelling tool may have different modes of interaction. For example, Hazel talked about how she might work with her physical trainer to *create* a diet plan, but that her husband would need to be involved in actually *carrying it out*. Collaborators in carer roles were generally viewed as operating in a more privileged capacity, as they are *in the story* with the storyteller. John suggested that his wife might like to use such a tool to create her own story as a carer, which could then be linked with his own.

Practically, participants also saw storytelling tools as a single, central location to share their stories with others, making it easier to bring everyone up to date. For healthcare professionals, these personal details could provide ‘ways in’^14^ to understand their patients and what is important to them. However, participants' concern with privacy showed that they would still want tight control over how this information was shared, and in many cases might only want to share general rather than specific information. June, for example, discussed not wanting to share her story with professionals that were dismissive of her concerns.

### 4.2 Addressing inequality and power disparities

Participants also linked telling their stories to personal advocacy and addressing power imbalances. All of the participants discussed the difficulties of trying to make those around them (even close friends and family) understand the lived experience of their health. Some participants related scepticism to the stigma surrounding their conditions—this was particularly notable amongst participants who had ME, which is consistent with similar research.^36^ Participants also said they faced inequality due to factors such as age and social background. In many cases, participants felt that they understood their conditions better than the professionals they were working with, reinforcing the idea that patients may become experts based on their lived experiences.^8^, ^12^, ^31^

### 4.3 Future research

Digital design for health storytelling is a research area which has so far received little attention. The goal of this project was to understand patient requirements for building such tools and to co‐design future prototypes with patients. The phenomenological methodology allowed deep insight into the emotional aspects of health stories. However, working with a smaller group of participants means the results are highly focused.

Future work could be done to understand the perspectives of collaborators (e.g., healthcare professionals, friends, family and carers), and to understand how they might interact within a storytelling tool. Research could also be used to determine how requirements for a tool might vary across participant groups with different conditions, or from different backgrounds. For example, digital literacy levels and access would greatly affect the value of such tools and how they might be used.

Participants commented very positively about the experience of sharing and seeing the representations of their stories, again demonstrating the positive effects of health storytelling on personal well‐being.^3^, ^4^ This suggests that an alternate approach to future work might be reproducing or adapting the methodology used here for use in treatment and care.

## 5 CONCLUSION

Previous research has identified a need for health stories to be included within patients' medical records in a clinical context and has also shown the many benefits which story‐based care can bring. At the same time, there has been an interest in further understanding the stories of people with multimorbidity, which are complex and extend beyond the clinical frame. This project set out to understand the attitudes of people with multimorbidity towards health stories, as well as their goals for using health storytelling tools, using a phenomenological approach which draws from lived experience. The understanding of the participants' stories was developed through iterative analysis and fieldwork, culminating in individual prototypes that encapsulate each participant's health story as well as their priorities for a health storytelling tool.

The project findings shed light on the goals and challenges for people with multimorbidity in health storytelling. Overall, participants wanted to use storytelling tools to reflect and remember their experiences, support conversations about their health, and advocate for themselves against scepticism. This reflects other research on the complexity of multimorbidity, the difficulties of coordinating care, and also the stigma faced through health inequalities.

This study demonstrates the potential value of health storytelling tools, as well as illustrates how such tools could support self‐management and treatment. While the original intention of the research was to benefit digital designers and developers interested in creating storytelling tools, the findings are of potential interest to anyone working with people with multimorbidity. Future work can build on this research by exploring collaborative storytelling, requirements across different patient groups and understanding professional perspectives for tools which help patients curate their stories.

## CONFLICTS OF INTEREST

The authors declare no conflicts of interest.

## ETHICS STATEMENT

Ethics was approved through an internal review by The Glasgow School of Art.

## Supporting information

Supporting information.

Supporting information.

Supporting information.

## Data Availability

The data that support the findings of this study are available on request from the corresponding author. The data are not publicly available due to privacy or ethical restrictions.
