# Supplementary material for: Patient co‐design of digital health storytelling tools for multimorbidity: A phenomenological study
Source: Health Expect. 2022 Sep 27;25(6):3073–84. doi: 10.1111/hex.13614 (PMC9700153; doi:10.1111/hex.13614)
Supplement: Supplementary file 1 — Supporting information. [file HEX-25--s002.pdf]

Appendix A – Participant Information Sheet

see me
hear me
know me

See me, hear me, know me:

Digital interfaces for health storytelling

[redacted]

This information sheet will explain more about what this involves, so that you can decide whether to participate.

Please feel free to discuss this with others if you wish, and if you need any further information, please ask.

What is this project about?

People living with long-term conditions are asked to constantly share information about their health conditions in order to access support, a process which can be complex and emotionally challenging. These stories are vital to help professionals better understand the person's challenges and goals, and yet they are not currently part of our health records. My project looks at how a piece of software could be designed for recording and sharing health stories. I've asked patients living with more than one health condition to take part, to understand how these more complex health stories can be recorded.

How is this project being funded?

My Master of Research training is funded by the Digital Health & Care Institute (DHI), which is a Scottish Funding Council initiative between the Glasgow School of Art, The University of Strathclyde, and NHS 24. The DHI aims to bring together health, care and third sector professionals, academics, industry partners, and citizens to work together to develop innovative ideas to overcome health and social care challenges.

see me
hear me
know me

What does the project involve?

If you decide to participate, we will do three 1-to-1 sessions together remotely online. Each session will last from 1 to 1.5 hours.

1. In the first session, I will ask you to tell me about your health story. The amount of detail you choose to provide is up to you – you will never be asked to share information that you don't want to.

2. In the second session, we will create a visual representation of your health story using digital tools I will prepare in advance. After this session, I will use the ideas that we created together to design a prototype for a new digital tool to support people to make and share their health story.

3. In the third session, we will look at the prototype together. You will have the opportunity to try out the prototype and tell me your thoughts.

Anything that we design during our sessions together will be kept by myself for the purposes of my research. However, if you would like a digital copy of the output for your own use, I would be happy to provide one.

What tools will be used?

Our online sessions will be carried out using the video conferencing software Zoom (https://zoom.us/), using a secure Pro account. Video recordings of each session will be recorded and stored securely by myself.

For our collaborative work, we will be using an online tool called Miro (https://miro.com/). This tool creates an virtual whiteboard where we can both work together. The board will be kept secure and only shared with the two of us for the course of the work. After we have finished work I will further restrict sharing to only myself, to make sure your information is kept safe.

see me
hear me
know me

Do I have to take part?

No. Taking part is your decision and it is entirely voluntary. You can also withdraw at any time, without giving a reason. If you would like to be involved, you will be asked to give verbal consent confirming that you understand what is going to happen and are happy to participate.

Will my taking part be kept confidential?

Yes, your participation will be kept entirely confidential.

<u>What will not happen:</u>

- No identifiable images of you will be used in the research output (e.g. my thesis paper).
- Your name and personal details will not be disclosed. You will have the opportunity to select a pseudonym (fake name) by which you will be identified.

<u>What will happen:</u>

- Images of the health story we create will be used in the research output. These will be edited to obscure any personal or identifying details. You will have the opportunity to review these and indicate anything you wish to keep confidential.
- If you agree, a video recording will be made of our sessions. You may also choose to have only audio recorded of you, with no video. The recording will only be used by myself and my supervisors, and will not be made public. If still images are used from this recording, I will blur any faces and personal details to ensure they remain anonymous.
- Selected quotations from the transcript of our sessions may be used anonymously in the research ouput.

Any items which contain personal information (such as your consent form) will be kept securely on The Glasgow School of Art Campus.

see me
hear me
know me

What will happen to the results?

Any information collected during the research will be anonymised and identified using a pseudonym (fake name). This will be altered to remove and/or obscure any personal or identifying data.

If you change your mind about anything you have shared, you can ask me to omit that from the final output. You will also have an opportunity to review the final output and give consent before it goes into the published version.

Your anonymised information will be used in publications (e.g. my thesis). It may also be used in an anonymised form on The Glasgow School of Art or DHI's websites, conference papers, journal articles, lectures, and broadcasts as part of future research and teaching.

What will happen to my information?

Your personal information (such as your name and contact details) will be kept securely on The Glasgow School of Art campus, and will be destroyed 3 months after the completion of the project.

Any data produced during the project (such as recordings of you) will also be securely kept as part of the research data. Following The Glasgow School of Art's research guidelines, it will be kept for up to 1 year after the completion of my course.

What if I want to withdraw?

You can choose to withdraw from the project at any time, simply by notifying me that you wish to do so. If you decide to withdraw, any information regarding yourself and your participation will be securely destroyed, and your research data will not be used in any output.

see me
hear me
know me

Contact

For more information about this project, please contact:

[redacted]

What if something goes wrong?

If this study has harmed you in any way or if you wish to make a complaint about the conduct of the study you can contact GSA using the details below for further advice and information:

[redacted]

Thank you!

Thank you for reading this information sheet and for considering taking part in my research. Please keep this sheet for future reference

INNOVATION SCHOOL THE GLASGOW SCHOOL OF ART

DIGITAL HEALTH & CARE INSTITUTE
