# Supplementary material for: Patient co‐design of digital health storytelling tools for multimorbidity: A phenomenological study
Source: Health Expect. 2022 Sep 27;25(6):3073–84. doi: 10.1111/hex.13614 (PMC9700153; doi:10.1111/hex.13614)
Supplement: Supplementary file 2 — Supporting information. [file HEX-25--s001.docx]

# Appendix B – Interview Schedules

## Interview 1

**About you**

Tell me a bit about yourself.

- What pseudonym would you like to use?
- How old are you?
- Tell me about what conditions you are currently managing.
- What object did you decide to bring to the session? How does it represent your health story to you?

**Before the illness**

What was your life like before your current health conditions started?

**Then something happened to me**

- Can you tell me a bit about when you first found out that you might have *condition(s)*?
- Do you remember the first sign that you might have condition(s)?
- How were you feeling physically and emotionally?
- What was your life like at the time that you were diagnosed?

**After that, things/some things were not like before**

- What happened after you were diagnosed?
- Who did you talk to about your diagnosis? (both healthcare professionals and family/friends)
- ­How did people react to your diagnosis?
- How did that make you feel?

﻿**Now**

- What is your health like currently?
- How are you managing your condition(s)?
- Have (or haven’t) people around you helped you with your condition(s)?
- How has your life changed since you were first diagnosed?

﻿**Tomorrow**

When you think about the future, where do you see yourself?

﻿**My story**

How did it feel to tell me your health story?

## Interview 2

**Introduction to the session**

*Researcher introduces the online whiteboarding tool to the participant.*

**Review of concepts**

*Participant is shown a collection of ideas curated by the researcher which were inspired by their health story (Interview 1). Some of these are digital sketches created by the researcher, others summarise the research of others on themes discussed by the participant.*

Here are some ideas I have put together based on our interview.

- What do you like or dislike about these?
- What would you change?

**Brainstorming**

*Following the concept review, the participant is asked to brainstorm ideas about how a storytelling prototype based on their story might work.*

- **Audience:** who am I telling this story to?
- **Collaborators:** am I the only author, or are there other people who I would want to collaborate on this story with me (family, friends, healthcare professionals)?
- **Focus:** What do I want someone to understand about me after reading it? What am I trying to convey?
- **Content:** What information about myself would I include?

**Co-design**

*In the final activity, participants were given a set of visual components prepared by the researcher based on the concepts they had been shown previously. They also had the ability to add their own shapes and text using the whiteboarding tool, as well as draw freehand.*

*Using these, participants were invited to work with the researcher to co-create their own visualisations in the whiteboarding tool. We referred back to the ideas generated from the brainstorming activity to help guide the co-creation process.*

## Interview 3

*In the final interview, participants reviewed an online, interactive prototype for a storytelling tool. Each prototype was individually developed by the researcher based on the findings from their previous two interviews.*

**Tool and methodology**

- Do you feel the final prototype accurately represents your perspective on health storytelling? If not, what should be changed to make it reflect you better?
- Part of the goal of this project was to use your story to create the final prototype. Do you have any thoughts/reflections on this process? How does it feel to see your story expressed in this way?
- If a tool like this existed, would it be useful to you in helping to understand and articulate your health story (both to yourself and others)?

**Participant feedback**

- Overall, how would you describe your experience participating in this project?
- Is there anything in particular which you felt did or didn’t work, or that you think could be improved for next time (e.g. logistics)?
- Do you have any other comments?
