# Supplementary material for: Patient co‐design of digital health storytelling tools for multimorbidity: A phenomenological study
Source: Health Expect. 2022 Sep 27;25(6):3073–84. doi: 10.1111/hex.13614 (PMC9700153; doi:10.1111/hex.13614)
Supplement: Supplementary file 3 — Supporting information. [file HEX-25--s003.docx]

# Appendix C – Analysis and Process

## Narrative analysis – holistic and form

The holistic content and form of the interviews were first analysed using a series of prompts adapted from the health storytelling elements identified by Rita Charon^1^.

**Frame**

Describe the storyteller’s surroundings.

**Form**

- Identify the genre of health story^2,3^ (e.g. quest, battle, journey).
- What metaphors does the storyteller use?
- Does the storyteller allude to any other events or topics?
- What is the storyteller’s presentation throughout (speaking style, emotional affect, facial expressions)?

**Time**

- What order is the story told in?
- What time period does it cover?
- What is its pacing?

**Plot**

Identify and describe the main categories (themes) in the story’s plot – link to relevant overarching categories, if any.

**Desire**

What do I feel that the storyteller wants me to understand by telling me this story? Why do I think they participated in this project?

## Narrative analysis – categories

Each interview was broken down into a series of quotes highlighting the most important points from each. These were then grouped by the researcher into themes and sub-themes over several passes. This grouping was done first for each individual interview, and then for all the interviews together.

## Digital design

The digital visuals and prototypes developed by the researcher were done using the tool Sketch (<https://www.sketch.com/>), drawing from the researcher’s own practice and experience working as a digital designer.

1. Charon R. Narrative Medicine: *Honoring the Stories of Illness*. Oxford University Press; 2006.

2. Hunsaker Hawkins A. *Reconstructing Illness: Studies in Pathography*. 2nd ed. Purdue University Press; 1999.

3. Frank AW. *The Wounded Storyteller*. 2nd ed. The University of Chicago Press; 2013. doi:10.7208/chicago/9780226260037.001.0001
